# Calculation of recovery plasticity in multistage hot forging under isothermal conditions

**DOI:** 10.1186/s40064-016-3570-x

**Published:** 2016-10-26

**Authors:** Iaroslav G. Zhbankov, Alexander V. Perig, Leila I. Aliieva

**Affiliations:** 1 Metal Forming Department, Donbass State Engineering Academy, Shkadinova 72, Kramatorsk, 84313 Ukraine; 2 Manufacturing Processes and Automation Engineering Department, Donbass State Engineering Academy, Shkadinova 72, Kramatorsk, 84313 Ukraine

**Keywords:** Hot deformation, Plasticity, Plasticity recovery, Multistage forging, Pause

## Abstract

A widely used method for hot forming steels and alloys, especially heavy forging, is the process of multistage forging with pauses between stages. The well-known effect which accompanies multistage hot forging is metal plasticity recovery in comparison with monotonic deformation. A method which takes into consideration the recovery of plasticity in pauses between hot deformations of a billet under isothermal conditions is proposed. This method allows the prediction of billet forming limits as a function of deformation during the forging stage and the duration of the pause between the stages. This method takes into account the duration of pauses between deformations and the magnitude of subdivided deformations. A hot isothermal upsetting process with pauses was calculated by the proposed method. Results of the calculations have been confirmed with experimental data.

## Background

A widely used method for hot forming steels and alloys, especially heavy forging, is the process of multistage forging with pauses between stages (stress relaxation technique). During these pauses in the forging process, the stress relaxation in metal allows decreased forging force. This technique is used extensively in the upsetting process in heavy forging as it is one of the most energy-intensive operations. There has been considerable research concerning this technique. The works of Sokolov and Efimov (Ukraine) (Sokolov 2002; Efimov et al. 1992; Sakunai et al. 2014; Bang et al. 2003) have made significant contributions in this field.

Another important and well known effect which accompanies multistage hot forging is increased metal plasticity in comparison with monotonic deformation. First of all this effect was manifested and used in forging special steels and alloys. Russian researcher Dzugutov (1977) marked that multi-stage deformation is used for increasing the plasticity of ingots forged from special steels and alloys with low plasticity. Multistage heavy forging is characterized by the amount of deformation between the forging stages which is defined by the stroke of the forging hammer or hydraulic press ram and also by the pause time between stages. For low-plasticity metal, the number of forging stages must be increased and amount of deformation per stage must be decreased. Dzugutov explains the physical meaning of increasing plasticity in multistage hot forging by the fact that low strain in each forging stage allows the forming of metal with fewer obstacles to dislocation movement. Increasing the number of pauses promotes a more extensive relaxation of the forged metal. He also marked that reducing the strain rate and increasing the number of forging stages (decreasing deformation for one stage) promotes diffusion processes and plasticity recovery.

Dzugutov established experimentally that during forging of hydraulic press billets from pearlitic structural alloyed steel, a value of 30–60% of relative strain on one stage of multistage forging can be achieved. During forging of high alloyed steels with low plasticity, an acceptable value of relative of strain on one stage of forging would be 6–10%.

Dzugutov (1977) marked that for alloy *ZhS6KP* only multistage forging with a one-stage relative strain of 2–4% and frequent heating allowed the achievement of positive results and acceptable values of limiting strain. It was proposed to determine the value of relative strain on one stage of forging experimentally.

Legrand (1956) shows that using of multistage forging for alloys *EI319* and *EI437* resulted in significantly increasing plasticity. By Legrand’s data the multistage hot forging of materials can increase plasticity by 2–2.5 times.

Dzugutov (1977) refers to Pavlov’s data and marked that during one stage upsetting (monotonic) of a billet of heat-resistant material the fracture became 20% of the relative height strain and during multistage upsetting of 12 stages the total relative height strain can be increased to 70%.

**Fig. 1:**
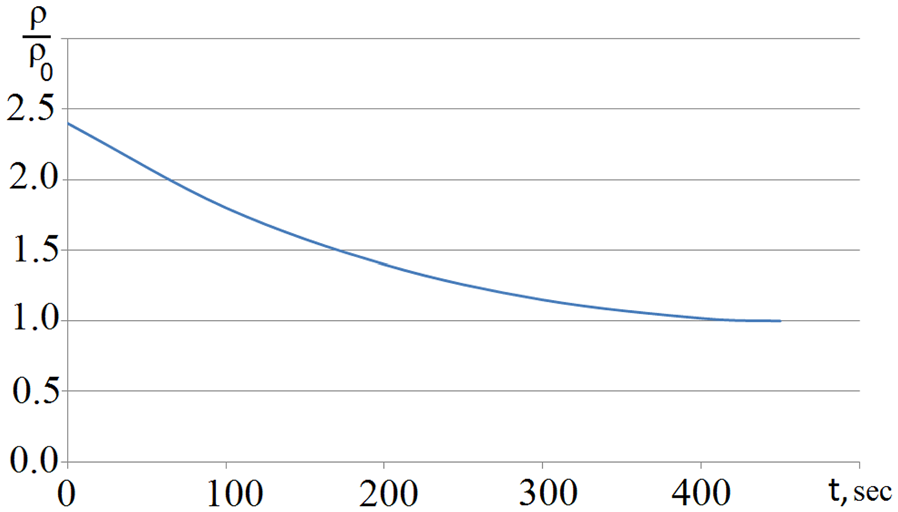
Influence of pause time on dislocation density in steel *1Kh18N9T* (temperature 1000 °C, relative strain 10%, where ρ and ρ_0_ are current (final) and initial dislocation densities)

Efimov established that during pauses in multistage forging the dislocation density in the forged billet decreases (Fig. 1). This regularity is the physical basis of increased plasticity and stress relaxation of the metal.

One of the types of multistage forging is vibration forging (upsetting) (Lin and Chen 2006). This type of forging allows increased uniformity of strain distribution in the billet and metal plasticity. Another type of multistage forging is deformation on rotary forging machines.

Based on the above, multistage forging shows promise for expanding hot forging to different steels and alloys. However, the absence of analytical description of these processes reduces their efficiency.

## Phenomenological analytical approach

The aim of this work is the development of an analytical method for the calculation of metal plasticity during isothermal multistage forging.

**Fig. 2:**
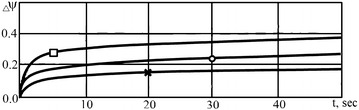
Recovery of plasticity steel *13Kh11N2V2MF-Ш* in pause after hot forging: *circle*, *cross*, *square*—T = 850, 900, 1100 °C

According to the data from Reference Mykhalevych (1998) the more intensive plasticity recovery occurs at the beginning of the pause. It was shown in Mykhalevych (1998) that further increases in pause duration leads to minor increases in plasticity recovery (Fig. 2).

Curves of recovery plasticity can be described by the next equation:

1 $$\Delta \psi = K(1 - e^{ - t \cdot n} ) ,$$

where *К*—coefficient which shows the maximum recovery of plasticity; *t*—pause duration, sec; *n*—coefficient which shows velocity of plasticity recovery.

Results of Eq. (1) match with those of the more complex equation (Mykhalevych 1998) when the pauses are small:

2 $$\Delta \psi = \frac{{\ln (1 + e^{{s\psi_{1} }} (e^{{s\Delta_{p} }} - 1))}}{s} - \Delta_{p} ,$$

where *s*—material coefficient; *Δ*_*p*_—relative duration of pause; *ψ*_1_—used plasticity before pause.

Equation (2) has a major fault with long pauses. This equation was obtained based on the hypothesis of full reversibility in the process of metal damage accumulation in the hot forging. The consequence of this hypothesis is that, during a long pause, the plasticity has a full recovery independent of the level of used plasticity.

In cold forging there is critical value of metal damage. After this damage level is reached, the metal cannot recover its initial plasticity after heat treatment (Matviychuk and Aliev 2009). These assumptions can be expressed for hot forging schemes. Suppose that maximum value of plasticity recovery (*Δψ*) with low used plasticity *ψ*_*i*_ on i-th stage of deformation is equal *ψ*_*i*_ and with a high value of used plasticity (close to 1) the recovery plasticity approaches 0. In this case the coefficient *K* of Eq. (1) takes the next form:

$$K = m \cdot \psi_{i} , and \left\{ {\begin{array}{*{20}c} {\psi_{i} = 0 \to m = 1} \\ {\psi_{i} = 1 \to m = 0} \\ \end{array} } \right.$$

**Fig. 3:**
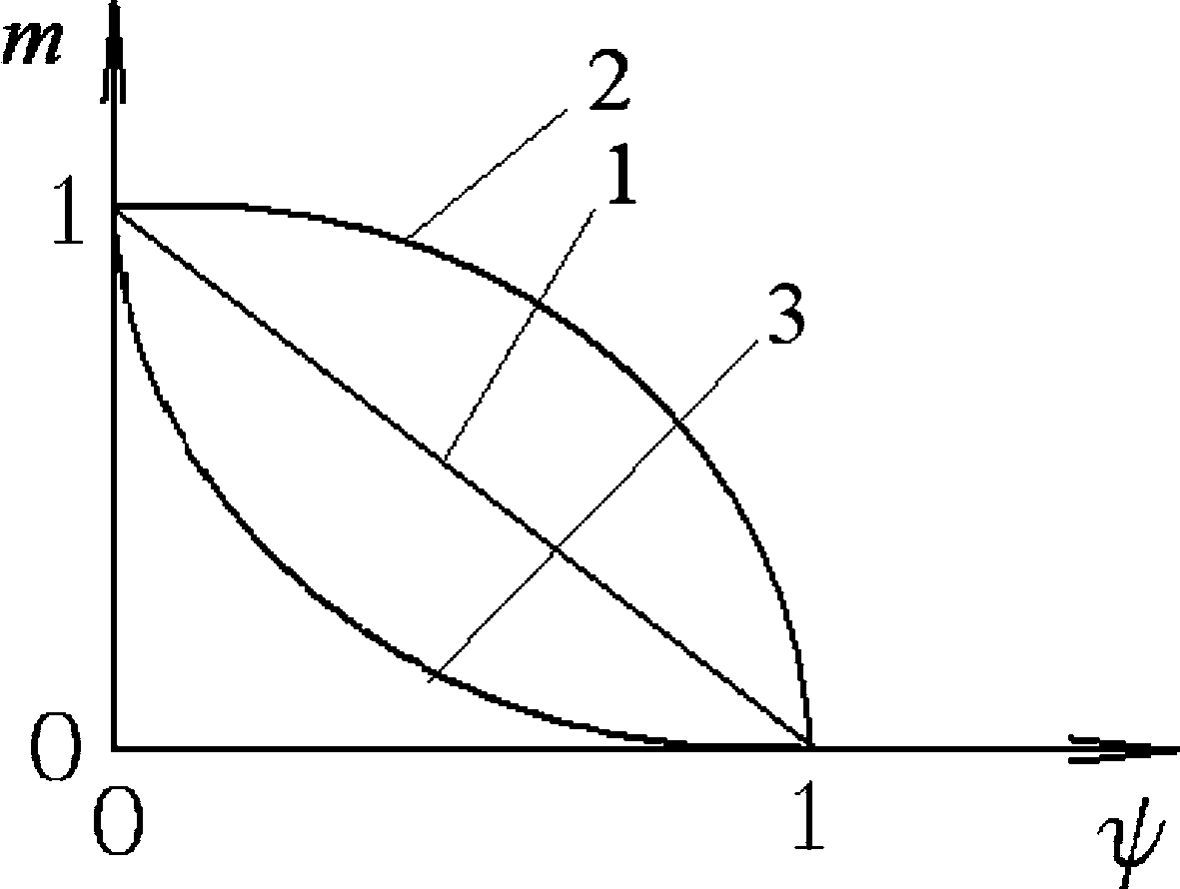
Dependence of coefficient m on the used plasticity of the previous stage (1—linear dependence *m* = 1 − *ψ*_*i*_; 2, 3—parabolic dependences $m = \sqrt {1 - \psi }_{i}$; *m* = (1 − *ψ*_*i*_)^2^)

Graphically it can be expressed by the next way (Fig. 3).

In this case Eq. (1) can be expressed as:

3 $$\Delta \psi = \psi_{i} \cdot m(1 - e^{ - t \cdot n} ) .$$

In general, parameters m and n are determined by material properties and deformation temperature. From the previous analysis it can be found that parameter *m* can be expressed as follows:

4 $$m = \left( {1 - \psi_{i} } \right)^{{f\left( {\psi_{i} ,\,T} \right)}} ,$$

where function *f*(*ψ*, *T*), characterizes the material, and can be found experimentally. To determine the function *f*(*ψ*, *T*) it is necessary to conduct a set of experiments. Specimens should be deformed under tensile conditions with certain prescribed temperature and velocity. Experiments should include deformation of the specimen with a value of *ψ*_*i*_ in the range of 0.1–0.9. This allows the determination of parameter *Δψ* and parameter m that simply define *f*(*ψ*, *T*) for Eq. (4). Equation (3) can be transformed to the following expression:

5 $$\Delta \psi = \psi_{i} \cdot \left( {1 - \psi_{i} } \right)^{{f\left( {\psi_{i} ,\,T} \right)}} \left( {1 - e^{ - t \cdot n} } \right)$$

Experimental determination of recovery plasticity value for calculation coefficients of Eq. (5) must be found by the next equation:

6 $$\Delta \psi = \psi_{1} + \psi_{2*} - 1 ,$$

where $\psi_{1} = {{\varepsilon_{u1} } \mathord{\left/ {\vphantom {{\varepsilon_{u1} } {\varepsilon_{p} }}} \right. \kern-0pt} {\varepsilon_{p} }}$—used plasticity before the pause; $\varepsilon_{p} = \ln \left( {{{l_{p} } \mathord{\left/ {\vphantom {{l_{p} } {l_{0} }}} \right. \kern-0pt} {l_{0} }}} \right)$—logarithmic strain fracture during monotonic tensile test; $\varepsilon_{u1} = \ln \left( {{{l_{l} } \mathord{\left/ {\vphantom {{l_{l} } {l_{1} l_{0} \cdot l_{0} }}} \right. \kern-0pt} {l_{1} l_{0} \cdot l_{0} }}} \right)$—logarithmic strain before the pause in the tensile test; $\varepsilon_{p2} = \ln \left( {{{l_{p2} } \mathord{\left/ {\vphantom {{l_{p2} } {_{0} }}} \right. \kern-0pt} {_{0} }}} \right)$—logarithmic strain of the fracture in the tensile test after the pause; $\psi_{2*} = \frac{{\varepsilon_{p2} - \varepsilon_{u1} }}{{\varepsilon_{p} }}$—residual plasticity after the pause; *l*_0_; *l*_1_—initial and pre-pause tensile test-derived lengths of the billet respectively, mm; $l_{p} ;\,l_{p2}$—maximum length of the billet in monotonic and multistage tensile test respectively, mm.

If recovery of plasticity does not occur i.e. *Δψ* = 0 then *ψ*_1_ + *ψ*_2*_ = 1 or

7 $$\frac{{\varepsilon_{p2} - \varepsilon_{u1} }}{{\varepsilon_{p} }} + \frac{{\varepsilon_{u1} }}{{\varepsilon_{p} }} = 1 .$$

In this case, the length of the fracture billet during the tensile test after the pause must be equal to the length of fracture billet during the monotonic tensile test without a pause $\varepsilon_{p2} = \varepsilon_{p}$.

Then Eq. (7) takes the next form:

$$\frac{{\varepsilon_{p} }}{{\varepsilon_{p} }} - \frac{{\varepsilon_{u1} }}{{\varepsilon_{p} }} + \frac{{\varepsilon_{u1} }}{{\varepsilon_{p} }} = 1$$

If all previously described equations are substituted in Eq. (6) and some mathematic simplification is done, we obtain:

$$\Delta \psi = \frac{{\ln \left( {\frac{{l_{p2} }}{{l_{0} }}} \right)}}{{\ln \frac{{l_{p} }}{{l_{o} }}}} - 1$$

**Fig. 4:**
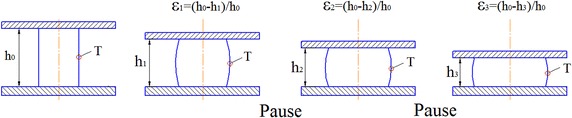
Scheme of a multistage upsetting process with intermediate pauses

A practical application of upsetting a cylindrical billet is shown in (Fig. 4). In the upsetting process point *T* is the point at which the fracture will most likely occur. Parameter *ψ*_1_ must be determined for this point. This parameter *ψ*_1_ depends on relative height deformation (ratio of the upsetting stroke to the initial billet height $\varepsilon_{h} = \Delta h/\Delta h_{0}$). At this point the values of logarithmic strain *ɛ*_*i*_ and used plasticity $\psi_{ 1} = \varepsilon_{ 1} /\varepsilon_{p}$ can be determined, where ɛ_p_—logarithmic strain of the fracture.

After the first stage of upsetting and pause the recovery of plasticity can be calculated by the next equation:

$$\Delta \psi = \psi_{1} \cdot \left( {1 - \psi_{1} } \right)^{{f\left( {\psi_{1} ,\,T} \right)\,}} \left( {1 - e^{ - t \cdot n} } \right)$$

Used plasticity after the first pause can be found as a difference between recovery plasticity at the end of pause and used plasticity before the pause:

$$\psi_{1}^{1} = \psi_{1} - \Delta \psi_{1}.$$

During the pause after the second stage of upsetting, plasticity will be recovered which was used during the second stage of upsetting *ψ*_2_ and some metal damage also accumulated after the first stage of upsetting. Assuming that used plasticity is additive (allowing the possibility of a simple summation) the value of recovery plasticity and used plasticity after two stages of upsetting can be found as follows:

$$\Delta \psi_{2} = \left( {\psi_{1}^{1} + \psi_{2} } \right) \cdot (1 - \psi_{1}^{1} - \psi_{2} )^{{f(\psi_{1}^{1} + \psi_{2} ,\,\,T)}} (1 - e^{ - t \cdot n} ); \psi_{2}^{1} = \psi_{1}^{1} + \psi_{2} - \Delta \psi_{2}$$

For the k stages of upsetting

8 $$\psi_{k}^{1} = \psi_{k - 1}^{1} + \psi_{k} - \Delta \psi_{k} ,$$

and $\psi_{k - 1}^{1} + \psi_{k} \le 1$, in another case recovery of plasticity is not calculated.

## Phenomenological numerical approach

For determination of the dependence of logarithmic strain *ɛ*_*i*_ at point T on the relative height deformation *ɛ*_*h*_ a simulation of the upsetting process was made. A finite element method (FEM) (Zhbankov and Perig 2013a, b; Zhbankov et al. 2014, 2016; Perig et al. 2013a, b, 2015) in Deform 3D software was used in the simulation of the upsetting process. The diameter of the billet is 18 mm, the length is 20 mm, and the material is carbon steel with 0.70% C. The initial temperature of the billet is 1100 °C and the initial temperature of the tools is 20 °C. The velocity of the upper tool motion is constant and equal to 1 mm/s. Tools in the simulation were rigid. The friction factor is 0.7 and the heat transfer coefficient is 5 W/(m^2^K). Materials simulation parameters are described in Table 1.

**Table 1:** Material simulation parameters

Simulation parameters	Values
Young’s modulus (GPa)	206
Poisson’s ratio	0.3
Yield function type	Von Mises
Hardening rule	Isotropic
Flow stress	$\overline{\sigma } = \overline{\sigma } (\overline{e} ,\mathop {\overline{e} }\limits^{ \bullet } ,T)$
Mesh number of billet	10,000
Minimum mesh width (mm)	0.7

**Fig. 5:**
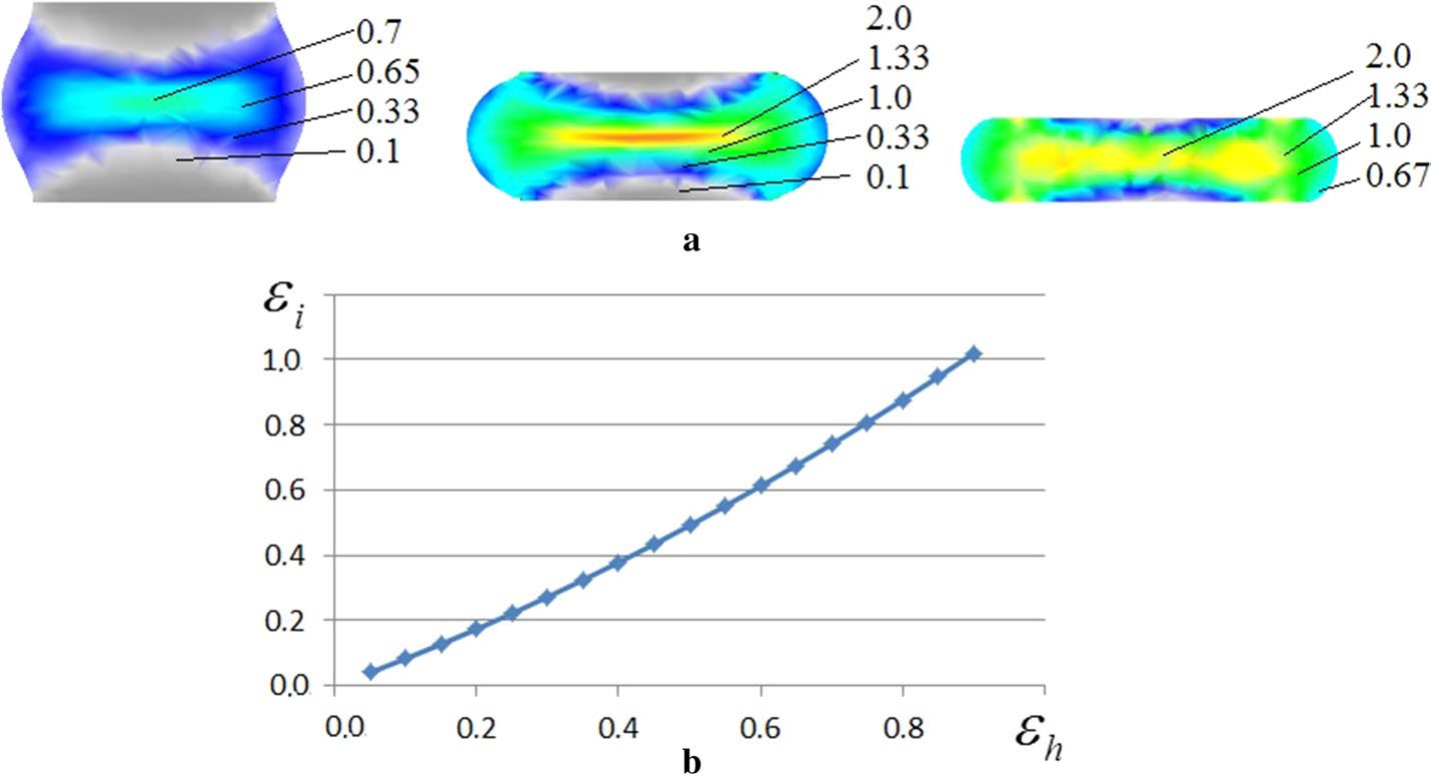
Distribution of equivalent plastic strain in the longitudinal section of the billet (**a**) and graphical dependence of the equivalent plastic strain on the relative height deformation of the billet (**b**)

Results of the simulation are fields of equivalent plastic strain in the longitudinal section of the billet and the graphical dependence of equivalent plastic strain on relative height deformation of the billet (Fig. 5).

After processing the obtained data, the analytical dependence of equivalent plastic strain on relative height deformation can be calculated, which is expressed for the given temperature and speed conditions in the form:

9 $$\varepsilon_{i} = 0.3725 \cdot \varepsilon_{h}^{2} + 0.7977 \cdot \varepsilon_{h} .$$

For the calculation of the maximum height deformation of the billet during multistage hot upsetting, the option of forging the billet from steel with 0.70% C in isothermal conditions with temperature 1100 °C, and strain rate 0.00066 s^−1^ was considered. By the data which was obtained experimentally by a monotonic isothermal tensile test of cylindrical billets with temperature 1100 °C, and strain rate 0.00066 s^−1^, the logarithmic strain in the fracture *ɛ*_*p*_ was equal to 0.65. We now assume that the stress state at the peripheral surface of the upset workpiece barrel is the same as the stress state in the tensile test. So the workpiece fracture in monotonic upsetting would occur at the instant, when the equivalent plastic strain at point T reaches the critical value of 0.65. This critical value of equivalent plastic strain at point T corresponds to a relative height deformation of the billet *ɛ*_*h*_ equal to 0.63, which could be found on the basis of Eq. (9). We now check the possibility of upsetting the billet to a higher relative height deformation through the introduction of multistage “fractional” forging. Now we estimate the multistage “fractional” forging of the billet with the “unit” height deformation in one stage *Δɛ*_*h*_ equal to 0.25 and the pause duration between stages 60 s on the basis of Eqs. (5–8).

Coefficient *m* is equal to 1 − *ψ*_*i*_ in Eq. (3) for a given temperature and material i.e. $f\left( {\psi_{i - 1}^{1} } \right) + \psi_{i} ,T$ for Eq. (5) is equal to 1 and parameter n has a value of 0.15 which follows from Fig. 2.

**Fig. 6:**
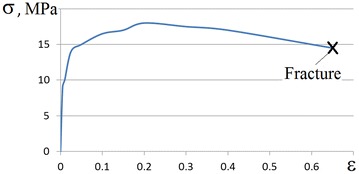
Stress-strain curve obtained by monotonic tensile of steel with 0.70% C (strain rate 0.00066 c^−1^, temperature 1100 °C)

**Fig. 7:**
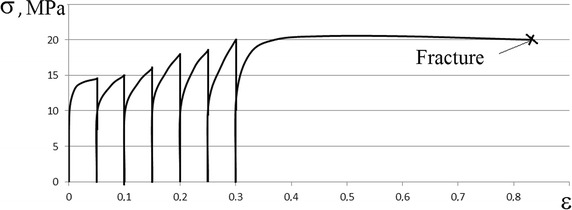
Stress-strain curve obtained by multistage tensile of steel with 0.70% C (strain rate 0.00066 c^−1^, temperature 1100 °C, pause duration 60 s)

Using the above relations and assumptions it was determined that multistage upsetting with one stage height deformation equal to 0.25 it is possible to increase the maximum height deformation from 0.63 to 0.75 for two stages with pauses for 60 s. Total height deformation in the upsetting can be increased by reducing deformation on one stage *Δɛ*_*h*_. Obtained results are confirmed by experimental data, which are described in Figs. 6, 7. Deviation of experimental data from the theoretical calculations is approximately 10%.

## Conclusions

A method for calculation of recovery plasticity in hot multistage forging has been proposed. This method allows the prediction of billet forming limits as a function of deformation during the forging stage and the duration of the pause between the stages.

A calculation of the isothermal multistage upsetting process of a cylindrical billet was made. This calculation shows that in monotonic upsetting the maximum relative height deformation is equal to 0.63. In multistage upsetting with relative height deformation in one stage equal to 0.25 and pause duration 60 s it is possible to increase the maximum height deformation to 0.75 without fracture.

## References

[CR1] Bang W, Lee CS, Chang YW (2003). Finite element analysis of hot forging with flow softening by dynamic recrystallization. J Mater Process Technol.

[CR2] Dzugutov MJa (1977) Plasticheskaja deformacija vysokolegirovannyh stalej i splavov [Plastic deformation of high alloyed steels and alloys]. Metallurgija [Metallurgy], Moscow (**in Russian**)

[CR3] Efimov VN, Sokolov LN, Savickij VV, Zhadkevich ML (1992) Vysokotemperaturnoe uprochnenie i razuprochnenie metallov i splavov [High temperature hardening and softening of metals and alloys]. Naukova dumka, Kiev (**in Russian**)

[CR4] Legrand SV (1956) Research on high-temperature alloys. Metallurgija [Metallurgy], Moscow (**in Russian**)

[CR5] Lin Zone-Ching, Chen Chun-kung (2006). Inverse calculation of the friction coefficient for upsetting a cylindrical mild steel by the experimental load. J Mater Process Technol.

[CR6] Matviychuk VA, Aliev IS (2009) Sovershenstvovanie processov lokal’noj rotacionnoj obrabotki davleniem na osnove analiza deformiruemosti metallov [Improving processes of local rotational forming based on analysis of deformability of metals]. DGMA [DSEA], Kramatorsk (**in Russian**)

[CR7] Mykhalevych VM (1998) Tenzorni modeli nakopychennia poshkodzhen [Tensor models of damage accumulation]. Universum-Vinnytsia, Vinnytsia (**in Ukrainian**)

[CR8] Perig AV, Zhbankov IG, Palamarchuk VA (2013). Effect of die radii on material waste during equal channel angular extrusion. Mater Manuf Processes.

[CR9] Perig AV, Zhbankov IG, Matveyev IA, Palamarchuk VA (2013). Shape effect of angular die external wall on strain unevenness during equal channel angular extrusion. Mater Manuf Processes.

[CR10] Perig AV, Tarasov AF, Zhbankov IG, Romanenko SN (2015). Effect of 2θ-punch shape on material waste during ECAE through a 2θ-die. Mater Manuf Processes.

[CR11] Sakunai Shingo, Okajima Takumo, Fujiwara Masakao, Otake Takuji (2014). Development of precise load prediction system for free forging of Ni-based superalloy having softening. Proced Eng.

[CR12] Sokolov LN (2002). Hardening—softening in open die forging. Improving processes and equipment of forming in mechanical engineering and metallurgy.

[CR13] Zhbankov IG, Perig AV (2013). Intensive shear deformation in billets during forging with specially formed anvils. Mater Manuf Processes.

[CR14] Zhbankov IG, Perig AV (2013). Forging of ingots without hot tops. Mater Manuf Processes.

[CR15] Zhbankov IG, Markov OE, Perig AV (2014). Rational parameters of profiled workpieces for an upsetting process. Int J Adv Manuf Technol.

[CR16] Zhbankov IG, Perig AV, Aliieva LI (2016). New schemes of forging plates, shafts, and discs. Int J Adv Manuf Technol.

